# Identifying a combined construct of grief and explosive anger as a response to injustice amongst survivors of mass conflict: A latent class analysis of data from Timor-Leste

**DOI:** 10.1371/journal.pone.0175019

**Published:** 2017-04-21

**Authors:** Susan J. Rees, Alvin Kuowei Tay, Elisa Savio, Zelia Maria Da Costa, Derrick Silove

**Affiliations:** 1 Psychiatry Research and Teaching Unit, University of New South Wales, Academic Mental Health Unit, Level 2 Mental Health Centre, The Liverpool Hospital, Sydney, Australia; 2 Alola Foundation, Dili, Timor-Leste; Moi University School of Medicine, KENYA

## Abstract

Previous studies have identified high rates of explosive anger amongst post-conflict populations including Timor-Leste. We sought to test whether explosive anger was integrally associated with symptoms of grief amongst the Timorese, a society that has experienced extensive conflict-related losses. In 2010 and 2011 we recruited adults (n = 2964), 18-years and older, living in an urban and a rural village in Timor-Leste. We applied latent class analysis to identify subpopulations based on symptoms of explosive anger and grief. The best fitting model comprised three classes: grief (24%), grief-anger (25%), and a low symptom group (51%). There were more women and urban dwellers in the grief and grief-anger classes compared to the reference class. Persons in the grief and grief-anger classes experienced higher rates of witnessing murder and atrocities and traumatic losses, ongoing poverty, and preoccupations with injustice for the two historical periods of conflict (the Indonesian occupation and the later internal conflict). Compared to the reference class, only the grief-anger class reported greater exposure to extreme deprivations during the conflict, ongoing family conflict, and preoccupations with injustice for contemporary times; and compared to the grief class, greater exposure to traumatic losses, poverty, family conflict and preoccupations with injustice for both the internal conflict and contemporary times. A substantial number of adults in this post-conflict country experienced a combined constellation of grief and explosive anger associated with extensive traumatic losses, deprivations, and preoccupations with injustice. Importantly, grief-anger may be linked to family conflict in this post-conflict environment.

## Introduction

Populations exposed to mass conflict and persecution commonly experience extensive losses [1, 2], experiences that are likely to provoke feelings of injustice and anger associated with symptoms of grief [3]. Yet there is a dearth of research investigating a possible nexus between grief and anger amongst populations living in post-conflict environments. We attempt to identify a subpopulation experiencing combined symptoms of grief and anger amongst survivors of prolonged persecution and conflict in Timor-Leste, and whether that putative pattern is associated with particularly high levels of traumatic loss, persisting preoccupations with injustice, and ongoing family conflict.

Anger as an unwanted and commonly dysfunctional emotional reaction has been associated with feelings of injustice amongst populations that have been exploited and persecuted. Having one’s human rights violated or economic goals systematically undermined can understandably lead to normal anger reactions, however, anger can also be associated with a loss of control, aggression and harm to others, including community members, intimate partners and children [4–10]. Anger has also long been regarded as a core component of the normal grieving process [11]. Moreover, clinical observations have suggested that a failure to resolve anger associated with a bereavement may contribute to the persistence of the grief reaction [12, 13], presumably because of strong feelings of grievance and injustice associated with the loss. In that regard, it is notable that studies examining the factorial structure of the persisting grief reaction have consistently identified anger and bitterness as core components [14, 15]. For example, a confirmatory factor analysis (CFA) conducted amongst bereaved adults in the USA identified anger/bitterness as one of six symptom domains of the construct of prolonged grief [14]. In keeping with this and other research, the constellation of anger-bitterness has been included in the categories of complex bereavement disorder (CBD) [16], defined as a diagnosis requiring further empirical evidence in DSM-5, as well as in the proposed ICD-11 definition of prolonged grief disorder (PGD) [17]. Nevertheless, controversy continues about the nosological status of these categories, particularly insofar as they distinguish pathological from normative forms of grief [18, 19].

Studies amongst post-conflict populations exposed to repeated traumatic losses may shed further light on the role of anger in the grief response. Our past research in Timor-Leste identified what appeared to be a high rate of explosive anger in response to trauma exposure. Explosive anger can express itself as physiological arousal and either verbal or physical aggression, the response characteristically being out of proportion to environmental triggers and experienced as uncontrollable, the subject reacting without immediate thought to the consequences [16]. Although in the aftermath of attacks, the person may feel a degree of relief or vindication, feelings of exhaustion, remorse and/or embarrassment are also common [20].

A population study in a rural and an urban village of Timor-Leste undertaken in 2004 recorded a prevalence of explosive anger of 38%, based on the international threshold of at least one attack of explosive anger a month (noting that the majority of these persons experienced much more frequent episodes) [9]. In a six-year follow-up study, the prevalence of explosive anger remained high (36%), suggesting that, at a population level, the reaction had a strong tendency to persist over a prolonged period of time [21]. Applying the stringent DSM-IV definition of intermittent explosive disorder (IED) which mandates the occurrence of acts of aggression in conjunction with anger, the prevalence if explosive anger was 8%, a high rate compared to other countries where the diagnosis has been studied at a population level [22–25]. A consistent finding of our studies in Timor-Leste is that women reported higher rates of explosive anger and IED than men, the converse of the usual gender pattern recorded in other countries [20, 22, 25]. Although a mixed methods study indicated that a range of experiences (exposure to conflict-related trauma and violent death of others, ongoing adversity, exposure to intimate partner violence) were associated with IED amongst women [20], these factors applied to other morbid mental health outcomes including post-traumatic stress disorder (PTSD) and depression, suggesting that the risk factors identified to date are not specific to anger [26, 27].

Doubts remain, therefore, about the origins and nature of explosive anger and its high prevalence in Timor-Leste, and why it is particularly common amongst women. In our endeavour to understand this phenomenon, we draw on the Adaptation and Development After Persecution and Trauma (ADAPT) model [28, 29] which highlights the core roles of interpersonal bond disruptions and experiences of injustice, amongst other domains, as major psychosocial challenges confronted by populations exposed to conflict. Although the model suggests that grief and anger represent the quintessential responses to disruptions in bonds and acts of injustice, respectively, these two experiences are likely to overlap given the inter-related nature and meaning of the traumatic events of conflict [29]. Specifically, traumatic losses are likely to occur in settings of gross injustices, thereby provoking simultaneous reactions of anger and grief. Other forms of adversity, for example conditions of material deprivation during and in the aftermath of conflict, may compound and prolong anger and grief. Symptoms of grief and anger in survivors of trauma may lead to ongoing conflict within families, representing one of the more severe longer-term psychosocial consequence of earlier exposure to mass violence [30].

The history of persecution and conflict in Timor-Leste provided a setting to investigate possible associations between grief and anger amongst a population exposed to extensive traumatic losses. The invasion and occupation of the territory by Indonesia in 1975 provoked a low-grade resistance war waged by members of the indigenous independence movement. During the period of conflict, which culminated in a humanitarian emergency in 1999, an estimated quarter of the indigenous population (of 600,000 persons at the time) died as a consequence of atrocities, warfare, burning of villages, murder, famine and untreated illness. In addition, there was widespread loss of property and livelihoods, and forced displacement of whole communities, with kinship and family groups being dispersed, some as refugees to other countries. In the post-conflict phase, further episodes of violence occurred, particularly in 2006–7, when a period of sustained internal conflict led to extensive injuries, deaths and displacement of communities into makeshift refugee camps. Socio-economic development in the newly independent country has been slow, with many families confronting extreme levels of poverty and deprivation.

Our aim was to test whether it is possible to identify a combined pattern of explosive anger and grief symptoms (grief-anger) amongst the Timorese population. We hypothesized that a subpopulation with grief-anger would report high levels of traumatic losses, preoccupations with injustice and ongoing adversity including family conflict in the post-conflict environment. We also examined whether women were more likely than men to experience the putative grief-anger constellation.

## Materials and methods

### Participants

Between June, 2010 and July, 2011, we conducted a survey of all adults, 18 years and older, living in every household in two administrative villages (sucos), one in Dili, the capital, the other, a rural site an hour’s drive away. Each suco is defined by contiguous hamlets (aldeias) falling under the administration of one chief (chefe). GPS and aerial mapping produced by the government for census purposes allowed us to identify all households in a setting where there is an absence of street names and many dwellings are located in remote wooded and mountainous areas. Both study sites were extensively affected by mass violence during the Indonesian occupation (1975–1999) and the subsequent internal conflict (2006–7).

### Field team and procedure

The team included 18 Timorese field workers with prior survey experience and/or psychology/public health degrees. They received a two-week training course followed by two months of field testing and piloting of survey measures under supervision. Pairs of interviewers were required to achieve a consistent 100 percent level of inter-rater reliability on the core measures. One hour long interviews were conducted in participants’ homes or another location if preferred by respondents, the procedure ensuring maximal privacy and confidentiality. In villages where families live in close proximity to each other, and where overcrowding is a problem, we sought to ensure privacy by taking participants to garden areas or away from the household to somewhere shaded and quite. We also arranged for children to be entertained by one of our colleagues if they were likely to cause a distraction to participants. Households were visited up to five times in order to meet potential participants.

### Ethics statement

The study was approved by the ethics committee of the University of New South Wales, the Ministry of Health of Timor-Leste, and the chiefs of each village. The majority of respondents gave written consent prior to commencement of interviews. Verbal consent was obtained in some cases where respondents were illiterate, trusted witnesses co-signing the forms. The procedure was endorsed by the community and received ethical approval from the University of New South Wales and the Ministry of Health of Timor-Leste.

### Measures

Our selection of constructs and the appropriate measures to assess them was based on theoretical considerations and the empirical findings in our past studies examining explosive anger in Timor-Leste.

The protocol, including the grief measure, was iteratively field tested amongst communities geographically adjacent and similar in sociodemographic composition to the sites of the definitive survey. In piloting, we applied an iterative process of feedback in which responses and solicited comments by respondents in the field were analysed and considered by a committee comprising Timorese of diverse backgrounds (age, gender, education, position in the community) and expatriate researchers. Measures were reviewed and revised to ensure that the constructs were understood by the community, items were readily comprehended, both semantically and linguistically, and response options (such as likert scales) were appropriately graduated according to the language and culture.

#### Exposure to conflict-related traumatic events

The 17 conflict-related traumatic events (TEs) listed in the Harvard Trauma Questionnaire (HTQ) [31] were modified to ensure their congruence with the historical context of Timor-Leste. TEs were recorded for two periods: the Indonesian occupation and the subsequent period (including the internal conflict) leading up to the study. We derived four broad TE domains based on their common nature and characteristics: conflict-related trauma, witnessing murder and atrocities, traumatic losses, and extreme deprivations (Table 1). Each TE item was scored 0–2, the maximum score being assigned if participants endorsed a TE for both time periods. We then generated a summary index for each of the four TE domains based on the addition of endorsed items.

**Table 1:** Socio-demographic characteristics of the sample (N = 2964).

	%	n
**Sex**		
Female	49	1451
Male	51.1	1513
**Location**		
Rural	62	1844
Urban	67.9	2013
**Mean age, year (sd)**	14.4	36.4
**Age group (years)**		
<24	19.5	578
25–34	34.3	1017
35–44	21.3	632
45–54	10.9	324
≥55	13.9	413
**Marital status**		
Married	67.9	2013
Single/never married	25.5	756
Widowed	5.8	171
Divorced	0.2	5
Separated	0.6	19
**Educational attainment**		
Completed primary	11.6	343
Completed junior high school	12.3	364
Completed senior high school	26.3	779
Completed tertiary	10.7	317
**Employment**		
Paid employment	34.8	1032
Subsistence farming/domestic duties/retired	43.1	1278
Unemployed	22.1	654
**Dimensions of injustice**		
Indonesian occupation	13.1	388
Internal conflict	24.6	729
Contemporary times	18.5	549
**Mental health outcomes**		
PTSD (2.2 threshold)	15.3	453
Severe distress	15.1	447
IED (DSM-IV criteria)	8.4	250
1 or more anger symptoms (range 0–5)	26.3	779
1 or more grief symptoms (range 0–4)	52.1	1544

#### Ongoing adversity

An inventory of daily adversities was developed based on extensive community consultations and refinement of items during piloting [20] (Table 2). All participants rated each adversity item on a five point scale (1 = not a problem, 2 = a bit of problem, 3 = moderately serious problem, 4 = a serious problem, 5 = a very serious problem). The adversity items were assigned to thematic domains: 1.poverty (insufficient food, lack of money for school fees and to meet traditional obligations to family, poor shelter, unemployment); 2. conflict with family (spouse, children, and extended family); and 3. conflict with community (with young people, and the wider community). The score for each domain was based on the summary score of constituent items (0 for lower levels of seriousness, 1 for moderate through to a very serious problem)

**Table 2:** Exposure to potential traumatic events.

Traumatic events	N	%	N	%	N	%
	Overall (n = 2964)		Men (n = 1451)		Women (n = 1513)	
**Conflict-related trauma (≥ 1 of the following events)**	1664	56.1	1039	71.6	625	41.3
Imprisonment	167	5.6	1038	71.5	1014	67
Involved in combat situation	298	10.1	251	17.3	230	15.2
Being physically assaulted	773	26.1	198	13.7	179	11.8
Torture	386	13	689	47.5	441	29.2
Involved in resistance movement	1323	44.6	78	5.4	36	2.4
**Witnessing murders and atrocities (≥ 1 of the following events)**	2344	79.1	1215	83.7	1129	74.6
Witnessing house burnt down	2052	69.2	156	10.8	11	0.7
Witnessing murder of family or friend	481	16.2	263	18.1	35	2.3
Witnessing murder of stranger	377	12.7	512	35.3	261	17.3
Witnessing someone being attacked	1130	38.1	322	22.2	64	4.2
Witnessing atrocities	114	3.9	868	59.8	455	30.1
**Traumatic losses (≥ 1 of the following events)**	1173	39.6	602	41.5	571	37.7
Forced separation from family members	529	17.9	216	14.9	313	20.7
Disappearance of family members	826	27.9	480	33.1	346	22.9
**Extreme deprivations (≥ 1 of the following events)**	2725	91.9	1340	92.4	1385	91.5
Deprivation of food or water	2638	89	1300	89.6	1338	88.4
No access to emergency medical care for self	1900	64.1	921	63.5	979	64.7
No access to emergency medical care for family	1914	64.6	919	63.3	995	65.8
Shortage of shelter	738	24.9	385	26.5	353	23.3

#### Preoccupations with injustice

Respondents were asked to identify and describe the worst human rights violation or other event associated with injustice they had experienced during three defined historical periods: the Indonesian occupation, the period of internal conflict, and contemporary time. Ratings were assigned as 1 for assigning an unjust event; 2 for experiencing preoccupations relating to the event; and 3 for distress related to these preoccupations. The composite index of injustice reflected the addition of scores for each of the three historical time periods (range 0–3).

#### Symptoms of explosive anger

Our community measure of explosive anger was developed, tested and modified serially during piloting to ensure its cultural appropriateness and comprehensibility in the local language, Tetum [32]. The screening questions inquired whether participants had ever experienced sudden episodes or attacks of anger and if so, how frequently these attacks occurred. Participants who endorsed attacks at a frequency of at least once a month were then asked about associated characteristics of loss of control, destruction of property, verbal aggression, and physical aggression towards others. We then applied an algorithm to derive a diagnosis of intermittent explosive disorder (IED) according to DSM-IV [32]. In a convergence study, we compared our community index of IED with a blinded diagnosis made on the Structured Clinical Interview for the Diagnostic and Statistical Manual for DSM-IV assigned by experienced psychologists [32]. There was a high level of concordance between the two measures: Area Under the Curve 0.90 (95% CI: 0.83–0.98). in the latent class analysis (described hereunder), we included the five core items of explosive anger as defined by IED each scored categorically (1 = present; 0 = absent): explosive anger attacks, loss of control of anger; destruction of property during attacks; verbal aggression during attacks; physical aggression towards others during attacks.

#### Grief symptoms

We inquired of all participants whether they had experienced a loss, defined as an event (since 1975) in which someone (e.g. family member, relative, or friend) close to the individual had died or been killed. Those who identified multiple losses were asked to identify the death that had the most impact on their lives, then recording the cause and time of the death. Almost all of these identified losses were related to traumatic deaths or untreated illness occurring during periods of mass conflict. Based on the identified loss event, participants were then asked to rate each of four grief items on a five-point frequency scale (0 = almost never, 1 = rarely, 2 = sometimes, 3 = often, 4 = always) as experienced in the past four weeks. The initial item pool was derived from the literature and contemporary criteria for assessing prolonged grief [17], the process of piloting reducing the number of symptoms to those that were widely recognised and regarded as core experiences of the Timorese people. The derived three symptom items were: persistent yearning/longing for the deceased, feelings of intense bitterness, and feelings emptiness in relation to the death. The fourth item assessed the level of functional impairment associated with the endorsed symptoms. For the latent class analysis, we assigned a score of 0 for symptoms scored not at all, rarely or sometimes and 1 if rated as often (3 on the scale) or always (4 on the scale).

#### Post-traumatic stress symptoms and psychological distress

Posttraumatic stress disorder (PTSD) symptoms and general symptoms of psychological distress (comprising depression, anxiety, somatic complaints) were assessed using the Harvard Trauma Questionnaire (HTQ) and Kessler-10 respectively, widely used measures applied in our previous studies in Timor-Leste [26]. In our aforementioned convergence study using the SCID, a satisfactory level of concordance was achieved for PTSD (AUC 0.82,95% CI: 0.71–0.94) and severe distress (compared to major depressive disorder) (AUC 0.79,CI, 0.67–0.91). A score of ≥ 2.2 for PTSD, and ≥ 30 for severe psychological distress (matching the international cut-off) produced the best balance between specificity and sensitivity for each index. Cronbach’s alpha for the PTSD scale was 0.95 and for the K10, 0.90.

All measures were translated into Tetum, the most widely spoken language in Timor-Leste. Minor inconsistencies were addressed during piloting and the final versions were translated and back-translated into English [33].

### Statistical analysis

We calculated intra-class correlations to assess for possible clustering within households of indices of grief, psychological distress, PTSD and explosive anger. All correlations were low (<0.05) indicating negligible clustering by households We used latent class analysis (LCA) to identify clusters of participants according to their pattern of symptoms of explosive anger and grief (each item scored in a binary manner as present or absent). We tested sequential models (one class, two classes, three classes, seriatim) examining a suite of conventional model fit indicators to assess for the best class solution: The Bayesian Information Criterion (BIC), sample size-adjusted Bayesian Information Criterion (SS-BIC), and the Akaike’s Information Criterion (AIC) [34, 35]. Lower values of these indicators indicate a better fit in comparing successive latent class models. In addition, we applied the Vuong-Lo-Mendell-Rubin (VLMR) and the Lo-Mendell-Rubin (LMR) adjusted likelihood ratio tests, both of which compare the fit of a latent class model of n classes to one with n+1 classes [36]. In judging the best-fitting model, we took into consideration the principle of parsimony, the degree of class separation, homogeneity of posterior probabilities within classes, and the interpretability of the classes yielded [35]. We draw on conventional criteria [37] in which conditional probabilities of 0.60 or above indicate a high probability of endorsing a particular symptom; values falling between 0.59 and 0.15, a moderate probability; and a value of 0.15 or less, a low probability.

After selecting the best-fitting model, we examined for associations between class membership (with the low symptom class as the reference category) and a range of relevant predictors using multinominal logistic regression analysis. The covariates included: sociodemographic characteristics of gender, residency in urban or rural areas, educational attainment, and employment; traumatic domains comprising conflict-related trauma, witnessing murders and atrocities, traumatic losses, and extreme deprivations; current adversities including indices of poverty, family conflict, and communal conflict; and preoccupations with injustice (during the Indonesian occupation, the internal conflict, and in contemporary times). Analyses were performed in STATA version 13 and Mplus Version 7.

## Results

### Sociodemographic characteristics

Of the 3368 respondents approached, 2964 (men, n = 1451, 49%; women, n = 1513, 51%) completed interviews, a response rate of 83.6% (inability to contact residents was by far the major reason for non-participation).

Table 1 indicates the socio-demographic characteristics of the sample. The mean age was 36.4 years (SD = 14.4), and a larger portion (n = 1844, 62%) resided in the rural area. Two-thirds (n = 2013, 67.9%) were married, the remainder being single/never married (n = 756, 25.5%), widowed (n = 171, 5.8%), divorced (n = 5, 0.2%) or separated (n = 19, 0.6%). In relation to education, 11.6% (n = 343) had completed primary, 12.3 (n = 364) junior, and 26.3 (n = 779) senior high school, whereas 10.7 (n = 317) had received post-school education (college/university). Nearly half (n = 1278, 43.1%) engaged in subsistence farming, domestic duties, or were retired; 34.8% (n = 1032) were occupied with paid employment (in a range of enterprises including government and private sectors); and the remainder were students or unemployed (n = 654; 22.1%).

### Prevalence of explosive anger, prolonged grief, PTSD, and severe distress

Two hundred and fifty persons (8.4%) met criteria for explosive anger according to IED criteria. A quarter (n = 779, 26.3%) endorsed one or more symptoms of explosive anger, including sudden anger attacks (n = 1074, 36.2%), loss of control (n = 662, 22.3%), verbal aggression (n = 637, 21.5%), destruction of property (n = 527, 17.8%), and physical aggression (n = 423, 14.3%).

Over half (n = 1544, 52.1%) endorsed one or more symptoms of prolonged grief, including persistent yearnings or longings for the deceased (n = 2178, 73.5%), feelings of bitterness about the death (n = 1293, 43.6%), and feelings of emptiness (n = 1152, 38.9%). A third (n = 957, 32.3%) reported functional impairment associated with these symptoms.

A similar number (n = 453, 15.3%) met the threshold for PTSD (>2.2) and severe psychological distress (n = 447, 15.1%) (≥30).

### Exposure to conflict-related traumatic events and ongoing adversity

Over half of participants (56.1%) reported experiencing one or more conflict-related traumas including political imprisonment, combat, physical assault, torture, and trauma related to involvement in the resistance movement (Table 2). Four out five persons reported witnessing murders and atrocities and two fifths traumatic losses, including forced separations and disappearances. Ninety percent experienced extreme deprivations related to access to urgent health care (for self or family), food, water and shelter.

### Ongoing adversity

Table 3 shows the frequency of adversity items. In order, poverty-related items endorsed were: shortage of electricity (n = 1983, 66.9%), no access to clean water (n = 1872, 63.2%), insufficient food (n = 1617, 54.6%) and money (n = 11586, 53.5%), problems accessing transport (n = 1489, 50.2%), environmental problems (n = 1527, 51.5%), lack of shelter (n = 1372, 46.3%), being unable to meet traditional family obligations (n = 1333, 45%); conflict with spouse (n = 446, 15.1) and extended family members (n- = 397, 13.4); youth conflict (n = 574, 19.4%); and safety issues in the community (n = 579, 19.5%).

**Table 3:** Exposure to ongoing adversities.

**Ongoing adversities**	**N**	**%**	**N**	**%**	**N**	**%**
	**Overall (n = 2964)**		**Men (n = 1451)**		**Women (n = 1513)**	
**Poverty**						
No access to healthcare	462	15.6	679	46.8	653	43.2
Insufficient food	1617	54.6	1179	81.3	1206	79.7
Insufficient money	1586	53.5	1295	89.3	1315	86.9
Insufficient money for education	1014	34.2	1050	72.4	1036	68.5
Insufficient money for traditional family obligations	1333	45	1157	79.7	1269	83.9
Being unable to fulfil familial expectations because of poverty	1059	35.7	1018	70.2	1089	72
Working too hard to support family	1045	35.3	843	58.1	1043	68.9
Poor shelter	1372	46.3	1116	76.9	1150	76
Unemployment	1192	40.2	1081	74.5	1033	68.3
No access to clean water	1872	63.2	1258	86.7	1310	86.6
Lack of electricity	1983	66.9	1354	93.3	1408	93.1
Lack of access to public transport	1489	50.2	1169	80.6	1274	84.2
Problems with surroundings	1527	51.5	1273	87.8	1359	89.8
**Family conflict**						
Conflict with husband or wife	446	15.1	500	34.5	567	37.5
Conflict with family members	397	13.4	500	34.5	531	35.1
**Communal conflict**						
Youth conflict	574	19.4	701	48.3	691	45.7
Problems (e.g. safety issues) in the community	579	19.5	692	47.7	760	50.2

### Preoccupations with past and present experiences of injustice

Distressing preoccupations with events associated with injustice were reported by 13.1% (n = 388) for the Indonesian occupation (1975–1999), 24.6% (n = 729) for the period surrounding the internal conflict (2002) and 18.5% (n = 549) in contemporary times (Table 1).

### Latent class analysis

Serial model testing concluded after assessing a four class LCA model (Table 4). Fit indicators improved up to the three-class model, the gains then being only marginal when progressing to a four class model. Importantly, the VLMR and the LMR adjusted likelihood ratio tests showed no statistical changes in progressing from a three to four class model. Given these findings and the ready interpretability of the classes, we adopted the three-class model.

**Table 4:** Goodness-of-fit statistics for latent class 1 to class 4 models.

**Model**	**VLMR**	**P**	**LMR**	**P**	
1 Class	-	-	-	-	
2 Class	3597.25	<0.0001	3552.8	<0.0001	
3 Class	1358.57	<0.0001	1341.78	<0.0001	
4 Class	581.09	0.31	573.91	0.32	
**Class**	**LR *χ***^**2**^	**BIC**	**SSABIC**	**AIC**	**Entropy**
1 Class	-15249	30569.9	30541.3	30515.9	-
2 Class	-13450	27052.6	26992.2	26938.7	0.84
3 Class	-12771	25774	25681.8	25600.1	0.81
4 Class	-12481	25272.8	25148.9	25039	0.8

Abbreviations: LR χ2: Likelihood Ratio Chi Square; AIC Akaike Information Criterion; BIC: Bayesian information Criterion, SSABIC: Sample Size Adjusted BIC; VLMR: Vuong-Lo-Mendell-Rubin likelihood ratio test; LMR: Lo-Mendell-Rubin adjusted likelihood ratio tests.

Table 5 shows the item probabilities for each class based on symptoms of grief and explosive anger. In the grief class (class 1, comprising 25% of the sample), item probabilities for preoccupations and bitterness were in the high probability range, and feelings of emptiness and functional impairment were in the moderate range. In contrast, all items of explosive anger items in this class fell into the low moderate or low probability range. In the combined explosive grief-anger class (class 2), comprising 24% of the sample, grief symptoms fell into the high (preoccupations) or moderate (bitterness, emptiness, functional impairment) ranges. In contrast to class 1, explosive anger symptoms fell into the high (episodes of explosive anger, verbal aggression) or high-moderate (loss of control, destruction of property, physical aggression) probability ranges. In the low symptom class (class 3), comprising 51% of the sample, there were low probabilities for the majority of symptoms of grief and explosive anger, with only two exceptions: preoccupations/yearning were in the moderate range and the generic item for explosive episodes was in the low/moderate range.

**Table 5:** Conditional probabilities for symptoms of explosive anger and grief based on a 3-class solution.

	Symptom		grief		Grief-anger		Low-symptom	
	Endorsement		(Class 1, 25%, n = 741)		(Class 2, 24%, n = 706)		(Class 3, 51%, n = 1517)	
**Symptoms of explosive anger**	%	n	Prob	SE	Prob	SE	Prob	SE
Explosive anger attacks	36.2	1074	0.26	0.03	0.82	0.02	0.19	0.02
Loss of control	22.3	662	0.17	0.03	0.66	0.03	0.04	0.01
Destruction of property	17.8	527	0.06	0.01	0.6	0.02	0.03	0.01
Verbal aggression	21.5	637	0.05	0.02	0.78	0.02	0.02	0.01
Physical aggression towards others	14.3	423	0.03	0.01	0.55	0.03	0.003	0.002
**Grief symptoms**								
Persistent yearning/longing for the deceased	75.5	2178	0.94	0.02	0.87	0.02	0.55	0.02
Feelings of bitterness	43.6	1293	0.86	0.04	0.6	0.03	0.12	0.02
Feelings of emptiness	38.9	1152	0.51	0.03	0.39	0.03	0.13	0.02
Functional impairment	32.3	957	0.68	0.04	0.41	0.03	0.08	0.01

### Comorbidity

In comparison to the low symptom class, both the grief and grief-anger classes were associated with PTSD (grief class: OR = 1.68, CI = 1.26–2.25; grief-anger class: OR = 1.99, CI = 1.49–2.67) and severe psychological distress (grief class: OR = 1.61, CI = 1.20–2.16; grief-anger class: OR = 2.42, CI = 1.82, 3.21).

### Associations with past trauma, ongoing adversity and preoccupations with injustice

Table 6 presents the findings of the multinomial logistic regression analysis testing for associations between the designated covariates (trauma, ongoing adversity, preoccupations with injustice) and the LCA classes.

**Table 6:** Multinomial logistic regressions examining predictors of latent class membership including indices of ptsd and severe psychological distress as covariates.

	Multivariate analysis		Multivariate analysis	
	Class 3 (low-symptom) as reference class		Class 2 (grief-anger) as reference class	
Predictors of class membership	OR (95% CI)	P-value	OR (95% CI)	P-value
**Sociodemographic characteristics**				
Residing in urban areas				
Class 1 (grief)	1.28 (1.02–1.60)	0.03	1 (reference)	
Class 2 (grief-anger)	1.53 (1.19–1.97)	0.001	1.19 (0.91–1.56)	0.21
Class 3 (low-symptom)	1 (reference)			
Sex (female)				
Class 1 (grief)	1.88 (1.52–2.31)	<0.000	1 (reference)	
Class 2 (grief-anger)	1.66 (1.32–2.08)	<0.000	0.86 (0.68–1.10)	0.22
Class 3 (low-symptom)	1 (reference)			
Educational attainment				
Completed primary school				
Class 1 (grief)	1.36 (0.89–2.08)	0.15	1 (reference)	
Class 2 (grief-anger)	1.34 (0.85–2.11)	0.20	0.92 (0.56–1.52)	0.74
Class 3 (low-symptom)	1 (reference)			
Completed junior high school				
Class 1 (grief)	1.06 (0.70–1.60)	0.80	1 (reference)	
Class 2 (grief-anger)	1.07 (0.67–1.68)	0.79	0.98 (0.59–1.63)	0.94
Class 3 (low-symptom)	1 (reference)			
Complete senior high school				
Class 1 (grief)	1.29 (0.90–1.83)	0.16	1 (reference)	
Class 2 (grief-anger)	1.11 (0.75–1.65)	0.59	0.85 (0.55–1.32)	0.48
Class 3 (low-symptom)	1 (reference)			
Unemployment				
Class 1 (grief)	0.88 (0.70–1.10)	0.25	1 (reference)	
Class 2 (grief-anger)	1.09 (0.86–1.38)	0.49	1.27 (0.98–1.63)	0.07
Class 3 (low-symptom)	1 (reference)			
**Traumatic events**				
Conflict-related trauma				
Class 1 (grief)	0.92 (0.74–1.51)	0.48	1 (reference)	
Class 2 (grief-anger)	0.99 (0.78–1.26)	0.94	1.09 (0.84–1.42)	0.51
Class 3 (low-symptom)	1 (reference)			
Witnessing murders and atrocities				
Class 1 (grief)	1.50 (1.17–1.91)	0.001	1 (reference)	
Class 2 (grief-anger)	1.66 (1.24–2.22)	0.001	1.15 (0.82–1.60)	0.42
Class 3 (low-symptom)	1 (reference)			
Traumatic losses				
Class 1 (grief)	1.44 (1.18–1.76)	<0.000	1 (reference)	
Class 2 (grief-anger)	1.79 (1.44–2.22)	<0.000	1.27 (1.01–1.61)	0.04
Class 3 (low-symptom)	1 (reference)			
Extreme deprivations				
Class 1 (grief)	1.33 (0.92–1.93)	0.12	1 (reference)	
Class 2 (grief-anger)	1.79 (1.06–3.04)	0.03	1.19 (0.66–2.15)	0.57
Class 3 (low-symptom)	1 (reference)			
**Current adversities**				
Poverty				
Class 1 (grief)	1.09 (1.05–1.13)	<0.000	1 (reference)	
Class 2 (grief-anger)	1.26 (1.21–1.31)	<0.000	1.18 (1.13–1.23)	<0.000
Class 3 (low-symptom)	1 (reference)			
Family conflict				
Class 1 (grief)	0.90 (0.74–1.10)	0.31	1 (reference)	
Class 2 (grief-anger)	1.44 (1.21–1.72)	<0.000	1.72 (1.41–2.09)	<0.000
Class 3 (low-symptom)	1 (reference)			
Communal conflict				
Class 1 (grief)	0.90 (0.76–1.06)	0.22	1 (reference)	
Class 2 (grief-anger)	1.01 (0.86–1.19)	0.87	1.12 (0.94–1.34)	0.20
Class 3 (low-symptom)	1 (reference)			
**Dimensions of injustice**				
Past injustice (Indonesian occupation)				
Class 1 (grief)	1.31 (1.19–1.45)	<0.000	1 (reference)	
Class 2 (grief-anger)	1.15 (1.03–1.28)	<0.000	0.90 (0.80–1.00)	0.06
Class 3 (low-symptom)	1 (reference)			
Past injustice (internal conflict)				
Class 1 (grief)	1.24 (1.14–1.36)	<0.000	1 (reference)	
Class 2 (grief-anger)	1.32 (1.19–1.45)	<0.000	1.08 (0.98–1.19)	0.14
Class 3 (low-symptom)	1 (reference)			
Present experience of injustice				
Class 1 (grief)	1.03 (0.95–1.11)	0.45	1 (reference)	
Class 2 (grief-anger)	1.17 (1.07–1.27)	<0.000	1.14 (1.04–1.24)	0.004
Class 3 (low-symptom)	1 (reference)			

The category of those completed university/college/vocational degree was omitted with an OR of 1. Sociodemographic characteristic variable were dummy coded. The analysis included PTSD (cut-off ≥≥2.2) and severe psychological distress (cutoff ≥30) as covariates. In comparison to the low-symptom class, both the grief (OR = 1.68, CI = 1.26–2.25) and grief-anger (OR = 1.99, CI = 1.49–2.67) classes were associated with PTSD and severe psychological distress (grief class: OR = 1.61, CI = 1.20–2.16; grief-anger class: OR = 2.42, CI = 1.82, 3.21).

In comparison to the low symptom reference class, women and urban dwellers were more likely to be assigned to both the grief and grief-anger classes. The two TE domains of witnessing murder and atrocities, and traumatic losses were both associated with the grief and grief-anger classes (relative to the reference class). In addition, however, the grief-anger class reported greater exposure to traumatic losses than the grief class. Also, the grief-anger class alone reported greater exposure to extreme deprivations related to conflict in comparison to the reference class. In relation to ongoing adversities, both the grief and grief-anger classes exceeded the reference class on the index of poverty; the grief-anger class in turn reported higher rates of poverty than the grief class. Only the grief-anger class reported greater levels of family conflict, in comparisons with both the reference low symptom class and the grief class. Compared to the low symptom class, both the grief and grief-anger classes reported greater preoccupations with injustice for the two historical periods of conflict (the Indonesian occupation and the later internal conflict). Only the grief-anger class, however, reported a higher level of preoccupations with injustice for contemporary times compared to the reference class.

## Discussion

Our analysis in post-conflict Timor-Leste, identified a typology comprising three subpopulations including those experiencing grief, grief-anger and low symptoms, the first two categories affecting a quarter of adults in the sample. Women and urban-dwellers were more likely to be assigned to both the grief and grief-anger classes. Compared to the low symptom reference class, both the grief and grief-anger classes reported greater exposure to conflict-related murders/atrocities and traumatic losses, more extreme levels of poverty, and distressing preoccupations with injustice related to two successive historical periods of conflict. There were important distinctions between the two morbid classes however, in that the grief-anger class reported greater exposure to traumatic losses (compared to the grief class), greater deprivations during the period of conflict (compared to the reference low symptom class), higher stress levels related to poverty (compared to the grief class), ongoing family conflict (compared to both the reference and grief classes), and preoccupations with injustice for contemporary times (compared to the reference and grief class).

Prior to discussing our findings, we consider the strengths and limitations of the study. The sample is one of the largest in the contemporary post-conflict mental health field and we achieved a high response rate. Although sampling was restricted to two localities, the sites were identified initially as being broadly representative of the socio-demographic profile of Timor-Leste as a whole [38]. Nevertheless, replication of the study in other areas of Timor-Leste and in post-conflict countries worldwide will be needed to test the generalizability of our findings. Caution needs to be exercised in inferring causal relationships from cross-sectional data of this kind. Longitudinal studies may assist in delineating the chronological sequencing of the relevant symptom constellations, in particular, whether anger precedes and thereby acts to prolong symptoms of grief. Recall of traumatic events can be subject to amnestic bias, although there was a notable consistency in the pattern of traumas documented and the known history of Timor-Leste. A systematic approach was followed in the transcultural adaptation, translation and testing of measures. Although the majority of losses identified as triggers of grief symptoms occurred several years earlier, our measure did not record the course of grief symptoms (whether fluctuating or chronic) so that judgement is reserved as to whether the reaction was prolonged or not.

Caveats notwithstanding, our findings cast new light on the high prevalence of explosive anger previously identified in community samples in Timor Leste [5, 9, 20], a phenomenon that has yet to be fully unexplained [20]. Even though previous studies had shown associations between explosive anger and common stressors such as conflict, poverty and injustice, these factors were common to other patterns of mental distress including symptoms of PTSD and severe psychological distress. Yet there were reasons to suspect that explosive anger had distinctive (albeit unidentified) antecedents given that the reaction appeared to be relatively independent as a construct from those of PTSD and severe psychological distress. In that regard, the identification of a subpopulation comprising a quarter of the sample that manifested the constellation of grief-anger offers a potential explanation for the high prevalence of anger identified in this society. Notably, although a grief class (with low anger symptoms) of equal size emerged, there was no independent explosive anger class, further accentuating the close association between anger with grief.

The grief-anger class reported the greatest exposure to traumatic losses, an important finding given that murder, atrocities and death by untreated illness and famine were widespread during the prolonged period of conflict in Timor-Leste. It may be that in collectivist societies such as Timor-Leste, losses that provoke strong and enduring feelings of injustice are particularly potent in generating the identified combined pattern of grief-anger. To confirm this, replication of our findings will be needed in other conflict-affected settings where traditional family and community values prevail.

Importantly, our regression analysis involving relevant covariates added credibility to the distinction we found between the grief-anger and grief classes. Specifically, the grief-anger class stood out in reporting high levels of traumatic loss, extreme deprivations during the period of conflict, severe ongoing poverty and family conflict, and preoccupations with injustice extending over three contiguous historical periods. In relation to the latter finding, we have reported a similar association between the anger component of persistent complex bereavement (PCB) disorder and a sense of injustice amongst refugees from West Papua, a neighbouring territory that has experienced a comparable level of prolonged mass conflict under Indonesian occupation [3].

The finding that a half of the population experienced relatively low levels of grief and anger symptoms offers some insights into the factors that protect post-conflict populations from these adverse psychological outcomes. It is notable that the low symptom group reported a similar level of exposure to the general traumas of conflict, indicating that they had not been sheltered from these events. It was only in the TE domains of witnessing murder/atrocities and traumatic losses that the low symptom group reported lower exposure, suggesting that protection from these salient forms of trauma may act to avert risk of developing the specific grief-anger constellation. Being male, living in a rural environment, experiencing lower levels of poverty and not experiencing family conflict were other factors that appeared protective, noting however, that cause-effect relationships involved remain to be confirmed given the cross-sectional nature of the study.

Our findings have potential implications for the individual, the family and the society as a whole, not only in Timor-Leste but in other post-conflict settings worldwide. In particular, confirmation of a grief-anger class and the social factors associated with the pattern, has the potential to add support to a cycles of violence model which postulates that exposure to the traumas of past conflict (in this instance, specifically traumatic losses and deprivations) may contribute to risk of subsequent family conflict in the aftermath of the violence [30]. We note however, that explosive anger associated with grief may be both a cause and a consequence of family conflict, resulting in a complex reciprocal and interacting effect that generates a vicious cycle of instability in the household. Our past qualitative data indicated that Timorese women with IED frequently recognized that their explosive anger led to harsh parenting behaviours which in some instances had an adverse effect on the health and well-being of their children [20]. It is possible therefore, that the grief-anger pattern we have identified contributes to the transgenerational transmission of trauma in a manner that impacts adversely on the psychosocial development of the next generation.

In relation to ongoing adversities, there appeared to be a stepwise relationship between the severity of poverty and the grief-anger, grief and low symptom classes respectively. These observations underscore the interaction between trauma-related mental health problems and socio-economic factors in post-conflict societies. Poverty places stress on individuals, families and communities, compounding past interpersonal and material losses in generating a sense of injustice and anger. In that sense, apart from the immediate hardship incurred by poverty, conditions of extreme material deprivations jeopardise recovery from trauma-related mental health conditions which in turn can impair functioning and reduce the capacity of survivors to engage in gainful employment or other opportunities to improve their economic well-being. [39].

## Conclusions

Our study identified a grief-anger constellation comprising a quarter of the study sample in post-conflict Timor-Leste. There were commonalities with the grief group in reporting greater exposure to witnessing murder, traumatic losses and poverty, and experiencing persisting preoccupations with injustice related to two consecutive historical periods of conflict. The grief-anger group was unique however, in reporting extreme levels of traumatic losses, exposure to material deprivations during the period of conflict, preoccupations with injustice in contemporary times and ongoing family conflict. It is a cruel irony that the traumatic rupture of interpersonal bonds during periods of mass conflict can generate a psychological reaction pattern (grief-anger) in survivors which in turn may undermine the survivor’s capacity to achieve a stable family environment in the post-conflict period.

## Supporting information

S1 Dataset This is the S1 Dataset. (DTA)

## References

[1] Başoğlu M, Livanou M, Crnobarić C, Frančišković T, Suljić E, Đurić D, et al Psychiatric and Cognitive Effects of War in Former Yugoslavia. JAMA. 2005;294(5):580–90. doi: 10.1001/jama.294.5.580 16077052 10.1001/jama.294.5.580

[2] Momartin S, Silove D, Manicavasagar V, Steel Z. Complicated grief in Bosnian refugees: Associations with posttraumatic stress disorder and depression. Comprehensive Psychiatry. 2004;45(6):475–82. doi: 10.1016/j.comppsych.2004.07.013 15526259 10.1016/j.comppsych.2004.07.013

[3] Tay AK, Rees S, Chen J, Kareth M, Silove D. Factorial structure of complicated grief: associations with loss-related traumatic events and psychosocial impacts of mass conflict amongst West Papuan refugees. Soc Psychiatry Psychiatr Epidemiol. 2015. Epub 2015/08/01. 10.1007/s00127-015-1099-x 26228854

[4] Berkowitz L, Harmon-Jones E. Toward an understanding of the determinants of anger. Emotion. 2004;4(2):107–30. Epub 2004/06/30. doi: 10.1037/1528-3542.4.2.107 15222847 10.1037/1528-3542.4.2.107

[5] Brooks R, Silove D, Steel Z, Steel CB, Rees S. Explosive anger in postconflict Timor Leste: Interaction of socio-economic disadvantage and past human rights-related trauma. J Affect Disord. 2011;131(1–3):268–76. doi: 10.1016/j.jad.2010.12.020 21310496 10.1016/j.jad.2010.12.020

[6] Fava M, Rosenbaum JF. Anger attacks in patients with depression. J Clin Psychiatry. 1999;60 Suppl 15:21–4. Epub 1999/07/27. PubMed PMID: 10418810. 10418810

[7] Hinton, Hsia C, Um K, Otto MW. Anger-associated panic attacks in Cambodian refugees with PTSD; a multiple baseline examination of clinical data. Behav Res Ther. 2003;41(6):647–54. 12732373 10.1016/s0005-7967(02)00035-9

[8] Rogler LH, Cortes DE, Malgady RG. The mental health relevance of idioms of distress. Anger and perceptions of injustice among New York Puerto Ricans. J Nerv Ment Dis. 1994;182(6):327–30. 8201304 10.1097/00005053-199406000-00003

[9] Silove D, Brooks R, Bateman Steel CR, Steel Z, Hewage K, Rodger J, et al Explosive anger as a response to human rights violations in post-conflict Timor-Leste. Soc Sci Med. 2009;69(5):670–7. doi: 10.1016/j.socscimed.2009.06.030 19616880 10.1016/j.socscimed.2009.06.030

[10] Eckhardt CI, Norlander B, Deffenbacher JL. The assessment of anger and hostility: a critical review. Aggression and Violent Behavior. 2004;9:17–43.

[11] Bonanno GA, Kaltman S. The varieties of grief experience. Clin Psychol Rev. 2001;20:1–30. 10.1016/s0272-7358(00)00062-3 11434227

[12] Parkes CM, Prigerson HG. Bereavement: Studies of grief in adult life: Routledge; 2013.

[13] Shear K, Shair H. Attachment, loss, and complicated grief. Developmental Psychobiology. 2005;47(3):253–67. doi: 10.1002/dev.20091 16252293 10.1002/dev.20091

[14] Simon NM, Wall MM, Keshaviah A, Dryman MT, LeBlanc NJ, Shear MK. Informing the symptom profile of complicated grief. Depress Anxiety. 2011;28(2):118–26. Epub 2011/02/02. PubMed Central PMCID: PMCPMC3079952. doi: 10.1002/da.20775 21284064 10.1002/da.20775 PMC3079952

[15] Prigerson HG, Shear MK, Jacobs SC, Reynolds CF 3rd, Maciejewski PK, Davidson JR, et al Consensus criteria for traumatic grief. A preliminary empirical test. Br J Psychiatry. 1999;174:67–73. Epub 1999/04/22. PubMed PMID: 10211154. 10211154 10.1192/bjp.174.1.67

[16] American Psychiatric Association. Diagnostic and Statistical Manual of Mental Disorders: Fifth Edition Washington, DC: American Psychiatric Association Press, 2013.

[17] Maercker A, Brewin CR, Bryant RA, Cloitre M, van Ommeren M, Jones LM, et al Diagnosis and classification of disorders specifically associated with stress: proposals for ICD-11. World psychiatry: official journal of the World Psychiatric Association (WPA). 2013;12(3):198–206. Epub 2013/10/08. PubMed Central PMCID: PMCPMC3799241. 24096776 10.1002/wps.20057 PMC3799241

[18] Bryant RA. Is pathological grief lasting more than 12 months grief or depression? Current opinion in psychiatry. 2013;26(1):41–6. Epub 2012/12/01. doi: 10.1097/YCO.0b013e32835b2ca2 23196998 10.1097/YCO.0b013e32835b2ca2

[19] Bryant RA. Grief as a psychiatric disorder. Br J Psychiatry. 2012;201(1):9–10. Epub 2012/07/04. doi: 10.1192/bjp.bp.111.102889 22753851 10.1192/bjp.bp.111.102889

[20] Rees, Silove D, Verdial T, Tam N, Savio E, Fonseca Z, et al Intermittent explosive disorder amongst women in conflict affected Timor-Leste: associations with human rights trauma, ongoing violence, poverty, and injustice. PLoS One. 2013;8(8):e69207 Epub 2013/08/21. PubMed Central PMCID: PMCPMC3737215. doi: 10.1371/journal.pone.0069207 23950885 10.1371/journal.pone.0069207 PMC3737215

[21] Silove D, Ivancic L, Rees S, Bateman-Steel C, Steel Z. Clustering of symptoms of mental disorder in the medium-term following conflict: An epidemiological study in Timor-Leste. Psychiatry Res. 2014;219(2):341–6. Epub 2014/06/17. doi: 10.1016/j.psychres.2014.05.043 24930578 10.1016/j.psychres.2014.05.043

[22] Kessler RC, Coccaro EF, Fava M, Jaeger S, Jin R, Walters E. The prevalence and correlates of DSM-IV intermittent explosive disorder in the national comorbidity survey replication. Arch Gen Psychiatry. 2006;63(6):669–78. doi: 10.1001/archpsyc.63.6.669 16754840 10.1001/archpsyc.63.6.669 PMC1924721

[23] Fincham D, Grimsrud A, Corrigall J, Williams DR, Seedat S, Stein DJ, et al Intermittent explosive disorder in South Africa: Prevalence, correlates and the role of traumatic exposures. Psychopathology. 2009;42(2):92–8. doi: 10.1159/000203341 19225243 10.1159/000203341 PMC3237393

[24] Al-Hamzawi A, Al-Diwan JK, Al-Hasnawi SM, Taib NI, Chatterji S, Hwang I, et al The prevalence and correlates of intermittent explosive disorder in Iraq. Acta Psychiatr Scand. 2012;126(3):219–28. Epub 2012/03/27. PubMed Central PMCID: PMCPMC3992890. doi: 10.1111/j.1600-0447.2012.01855.x 22443168 10.1111/j.1600-0447.2012.01855.x PMC3992890

[25] Yoshimasu K, Kawakami N. Epidemiological aspects of intermittent explosive disorder in Japan; prevalence and psychosocial comorbidity: findings from the World Mental Health Japan Survey 2002–2006. Psychiatry Res. 2011;186(2–3):384–9. Epub 2010/08/17. PubMed Central PMCID: PMCPMC3012136. doi: 10.1016/j.psychres.2010.07.018 20709410 10.1016/j.psychres.2010.07.018 PMC3012136

[26] Silove D, Liddell B, Rees S, Chey T, Nickerson A, Tam N, et al Effects of recurrent violence on post-traumatic stress disorder and severe distress in conflict-affected Timor-Leste: a 6-year longitudinal study. Lancet Glob Health. 2014;2(5):e293–300. Epub 2014/08/12. doi: 10.1016/S2214-109X(14)70196-2 25103168 10.1016/S2214-109X(14)70196-2

[27] Silove D, Brooks R, Bateman CS, Steel Z, Amaral ZF, Rodger J, et al Social and trauma-related pathways leading to psychological distress and functional limitations four years after the humanitarian emergency in Timor-Leste. J Trauma Stress. 2010;23(1):151–60. Epub 2010/02/11. doi: 10.1002/jts.20499 20146257 10.1002/jts.20499

[28] Silove D. The ADAPT model: a conceptual framework for mental health and psychosocial programming in post conflict settings Intervention. 2013;11.

[29] Silove D. The psychosocial effects of torture, mass human rights violations, and refugee trauma: Toward an integrated conceptual framework. J Nerv Ment Dis. 1999;187(4):200–7. 10221552 10.1097/00005053-199904000-00002

[30] Rees S, Thorpe R, Tol W, Fonseca M, Silove D. Testing a cycle of family violence model in conflict-affected, low-income countries: A qualitative study from Timor-Leste. Soc Sci Med. 2015;130:284–91. Epub 2015/03/11. doi: 10.1016/j.socscimed.2015.02.013 25753169 10.1016/j.socscimed.2015.02.013

[31] Mollica RF, Caspi-Yavin Y, Bollini P, Truong T, Tor S, Lavelle J. The Harvard Trauma Questionnaire: Validating a cross-cultural instrument for measuring torture, trauma, and posttraumatic stress disorder in Indochinese refugees. J Nerv Ment Dis. 1992;180(2):111–6. 1737972

[32] Liddell BJ, Silove D, Tay K, Tam N, Nickerson A, Brooks R, et al Achieving convergence between a community-based measure of explosive anger and a clinical interview for intermittent explosive disorder in Timor-Leste. J Affect Disord. 2013;150(3):1242–6. Epub 2013/07/10. doi: 10.1016/j.jad.2013.06.006 23835102 10.1016/j.jad.2013.06.006

[33] Van Ommeren M, Sharma B, Thapa S, Makaju R, Prasain D, Bhattarai R, et al Preparing instruments for transcultural research: Use of the translation monitoring form with Nepali-speaking Bhutanese refugees. Transcult Psychiatry. 1999;36(3):285–301. Export Date 12 February 2012.

[34] McGCutcheon AC. Latent class analysis. Beverly Hills: CA: Sage; 1987.

[35] Collins LM, Lanza ST. Latent Class and Latent Transition Analysis: With Applications in the Social, Behavioral, and Health Sciences: Wiley; 2009. 330 p.

[36] Nylund KL, Asparouhov T, Muthen B. Deciding on the number of classes in latent class analysis and growth mixture modeling: A monte carlo simulation study. Structural Equation Modeling. 2007;14(4):535–69.

[37] Burstein M, Georgiades K, Lamers F, Swanson SA, Cui L, He JP, et al Empirically derived subtypes of lifetime anxiety disorders: developmental and clinical correlates in U.S. adolescents. J Consult Clin Psychol. 2012;80(1):102–15. Epub 2011/11/16. PubMed Central PMCID: PMCPMC3265653. doi: 10.1037/a0026069 22081863 10.1037/a0026069 PMC3265653

[38] Silove D, Bateman CR, Brooks RT, Fonseca CA, Steel Z, Rodger J, et al Estimating clinically relevant mental disorders in a rural and an urban setting in postconflict Timor Leste. Arch Gen Psychiatry. 2008;65(10):1205–12. Epub 2008/10/08. doi: 10.1001/archpsyc.65.10.1205 18838637 10.1001/archpsyc.65.10.1205

[39] Cardozo BL, Bilukha OO, Crawford CA, Shaikh I, Wolfe MI, Gerber ML, et al Mental health, social functioning, and disability in postwar Afghanistan. JAMA. 2004;292(5):575–84. Epub 2004/08/05. doi: 10.1001/jama.292.5.575 15292083 10.1001/jama.292.5.575

